# Correlates of sexual initiation among European adolescents

**DOI:** 10.1371/journal.pone.0191451

**Published:** 2018-02-08

**Authors:** Pietro Gambadauro, Vladimir Carli, Gergö Hadlaczky, Marco Sarchiapone, Alan Apter, Judit Balazs, Raphaela Banzer, Julio Bobes, Romuald Brunner, Doina Cosman, Luca Farkas, Christian Haring, Christina W. Hoven, Michael Kaess, Jean Pierre Kahn, Elaine McMahon, Vita Postuvan, Merike Sisask, Airi Värnik, Nusa Zadravec Sedivy, Danuta Wasserman

**Affiliations:** 1 National Centre for Suicide Research and Prevention of Mental Ill-Health (NASP), Department of Learning, Informatics, Management and Ethics (LIME), Karolinska Institutet, Stockholm, Sweden; 2 Res Medica Sweden, Gynaecology and Reproductive Medicine, Uppsala, Sweden; 3 Department of Medicine and Health Science, University of Molise, Campobasso, Italy; 4 National Institute of Health for Migration and Poverty, Rome, Italy; 5 Schneider’s Children Medical Center of Israel, Tel Aviv University, Tel Aviv, Israel; 6 Vadaskert Child Psychiatric Hospital and Outpatient Clinic, Budapest, Hungary; 7 Institute of Psychology, Eötvös Loránd University, Budapest, Hungary; 8 Addiction Help Services BIN, Innsbruck, Austria; 9 Institute of Psychology, University of Innsbruck, Innsbruck, Austria; 10 Department of Psychiatry, University of Oviedo, CIBERSAM School of Medicine, Oviedo, Spain; 11 Department of Child & Adolescent Psychiatry, Center for Psychosocial Medicine, University of Heidelberg, Heidelberg, Germany; 12 Clinical Psychology Department, Iuliu Hatieganu University of Medicine and Pharmacy, Cluj-Napoca, Romania; 13 Psychiatry and Psychotherapy B, State Hospital Hall in Tyrol, Hall, Austria; 14 Department of Child and Adolescent Psychiatry, Columbia University-New York State Psychiatric Institute, New York, United States of America; 15 Department of Epidemiology, Mailman School of Public Health, Columbia University, New York, United States of America; 16 University Hospital of Child and Adolescent Psychiatry and Psychotherapy, University of Bern, Bern, Switzerland; 17 Department of Psychiatry and Clinical Psychology, CHRU de NANCY and Pôle 6, Centre Psychothérapique de Nancy-Laxou, Université de Lorraine, Nancy, France; 18 National Suicide Research Foundation, University College Cork, Cork, Ireland; 19 Slovene Center for Suicide Research, Andrej Marusic Institute, University of Primorska, Koper, Slovenia; 20 Estonian-Swedish Mental Health & Suicidology Institute, Tallinn, Estonia; 21 School of Governance, Law and Society (SOGOLAS), Tallinn University, Tallinn, Estonia; 22 School of Natural Sciences and Health, Tallinn University, Tallinn, Estonia; Universita Cattolica del Sacro Cuore Sede di Roma, ITALY

## Abstract

**Background:**

Sexuality is a physiological component of adolescent development, though early initiation is associated with reproductive health risk. This study aimed at identifying correlates and predictors of sexual initiation in a large multinational cohort of European adolescents.

**Methods:**

A questionnaire addressing socio-demographics, behaviours, mental health and sexual activity, was delivered to 11,110 adolescents recruited from 168 randomly selected schools in 10 European countries between 2009 and 2011. A follow-up questionnaire was delivered after 12 months. The longitudinal association of baseline risk behaviors, psychological attributes and contextual vulnerabilities, with sexual initiation during follow-up was evaluated through simple and multivariable age/sex stratified logistic regression. Multinomial logistic regression measured the association between predictors and sexual initiation with or without coexisting reproductive risk factors, such as multiple partners or infrequent condom use.

**Results:**

Baseline sexual experience was reported by 19.2% of 10,757 respondents (median age 15; IQR 14–15; females 59.6%). This was significantly more frequent among pupils older than 15 (41%) and males (20.8%). Of 7,111 pupils without previous experience who were available at follow-up (response rate 81.8%), 17% reported sexual initiation, without differences between females and males. Baseline smoking (age/sex adjusted odds ratio [aOR] 3.63), alcohol use (aOR 2.95), illegal drugs use (aOR 2.72), and poor sleep (aOR 1.71) predicted sexual initiation. Stratified analyses showed a particularly strong association in case of younger and female pupils, and, among girls, when initiation was reported together with multiple partners and/or infrequent condom use. Externalizing (i.e. conduct and hyperactivity) symptoms independently predicted sexual initiation. Internalizing difficulties (i.e. emotional and peer problems) were negatively associated with early and risky sexual initiation among boys. Significant predictors included also being bullied, fighting, truancy, and low parental involvement.

**Conclusions:**

Adolescent sexual behaviours are related to non-sexual risk behaviours, psychological difficulties and contextual vulnerabilities. While gateway effects explain some associations, a comprehensive model is needed to understand adolescent sexual behaviours, their physical, mental, and social health outcomes, and their potential positive effects on wellbeing. Tailored interventions may need to consider younger girls as a particularly vulnerable group in view of a strong association between non-sexual and sexual behaviors.

## Introduction

Behaviours and lifestyle choices shape adolescents’ current and future health. Risk-taking behaviours during adolescence, with their normative prevalences and frequent clustering [1], are therefore a major concern. In the context of adolescent risk-taking, sexual behaviours are peculiar. Sexuality is a normative and physiological component of adolescent development. Yet, earlier sexual initiation is associated with serious risks such as unwanted pregnancies and sexually transmitted diseases, which potentially lead to further consequences [2–5]. Those outcomes are often mediated by unsafe sexual practices, such as inconsistent contraception or multiple partners, which early initiators are more likely to maintain during adult age [6,7].

Further understanding of the mechanisms by which adolescent sexuality may become a risk behaviour, although paramount, faces obstacles. The definition of “early” sexual debut is inherently challenging and impossible to abstract from individual or contingent factors such as consent, awareness and access to safe sex, so that individual risk may not be represented accurately by the timing of initiation. Nevertheless, the obvious downstream relationship between sexuality and reproductive health risk commonly catalyzes policy efforts, which have traditionally focused on avoiding, postponing or limiting sexual activity, as preventive measures [3]. However, isolated responses, framing sexual initiation as a negative event because of the inherent reproductive health risk, are arguably inadequate because they do not account for the fullness of adolescents’ sexual behaviours and lives, and overlook the fact that sexuality may, and should, be a positive experience [8]. Furthermore, the common co-occurrence of multiple risks behaviours [9] and the potential mental and social health outcomes of adolescent sexuality [8] are often neglected by initiatives targeting exposures and outcomes directly related to the reproductive health domain. Such single-domain initiatives may therefore be ineffective towards adolescent health’s big picture.

As adolescent health is a global concern and risk behaviours’ clustering is cross-nationally consistent [10], joint transnational efforts facilitating the translation of research findings into common policies and practice are desirable but still difficult in wide geopolitical regions. Most large data proceed from national surveillance programmes, such as the American Youth Risk Behavior Surveillance System (YRBSS) [11]. No similar initiative covers specifically the European Union, although the World Health Organisation (WHO) has collected data about adolescents’ well-being through the Health Behaviour in School-aged Children (HBSC) project, a cross-sectional survey across 45 countries/regions of the WHO European region and North America [12]. Studies, supported by similar national or cross-national programmes, have described consistent associations between sexual and non-sexual risk behaviours [10,11], and a gateway effect has often been claimed as explanatory mechanism [9]. For instance, alcohol or illegal drugs may increase the opportunities for sexuality and sexual risk behaviours [13]; high media consumption facilitates exposure to sexual content or contacts with potential partners [14]; early sexual experiences may be forced and related to violence [15].

One limitation to gateway interpretations is that most available data are cross-sectional, while longitudinal studies are needed to make inferences about causality or show directionality [9]. Furthermore, the simplified explanations provided by gateway models may neglect the modulating effect of individual and contextual factors [16] and result into downstream policies targeting individual health problems by avoiding exposure to the gateway or minimizing related outcomes [3,9]. A more comprehensive perspective of behavioural risk syndrome would instead consider broader determinants or modulators, aside from risk behaviours and related health outcomes [9,10,17–19]. Individual psychological difficulties, for instance, are related to adolescent behaviour and its effect on wellbeing. Externalizing or internalizing symptoms are associated with different patterns of adolescent risk-taking [1]; however, they may also influence adolescents' evaluation of their behaviours and, consequently, mental and social health outcomes [8]. Even relationships with peers and family may influence both sexuality and its perception, which arguably mediates the effect of sexual behaviours on adolescent wellbeing [8]. Additionally, sex is a plausible modulator of the association between adolescent sexual behaviour and its correlates, as girls and boys have different patterns of engagement in risk behaviours [1] and may be influenced by double standards [20].

In such a complex context, our understanding of adolescent health would arguably benefit from longitudinal and cross-domain data about the correlates of risk behaviours. The main objective of this study was to identify correlates and predictors of sexual initiation among non-sexual risk behaviors, individual psychological attributes, and problematic contextual interactions, in a large and multinational cohort of European high school pupils. A secondary objective was to verify whether the association between predictors and sexual initiation changes depending on age, sex or the coexistence of sexual risk behaviours.

## Materials and methods

This study was conducted as a part of SEYLE (Saving and Empowering Young Lives in Europe), an EU-funded project designed to collect epidemiological data and to conduct a randomized trial of suicide-preventive interventions [21,22]. Between 2009 and 2011, 11,110 adolescents were recruited to the SEYLE trial from 168 randomly selected schools in 10 European countries: Austria, Estonia, France, Germany, Hungary, Ireland, Italy, Romania, Slovenia and Spain. The eligibility of schools and adolescents was determined by previously published criteria [21]. The sample size was based on the expected effect of the trial’s suicide-preventive interventions and calculated in excess of the requirements to detect statistically significant changes [21]. The sample had a high external validity and was fairly representative of each national population [23]. Further details on sample selection, size and randomization are elsewhere published [21].

Once the consent of pupils and their caregivers were obtained locally, a baseline self-report questionnaire addressing sociodemographics, behaviours, life-events, relations, and mental health, was completed during one classroom session. A follow-up questionnaire was delivered after 12 months. The questionnaires were prepared in English and underwent coordinated translation and cultural adaptation in each country [23].

**Fig 1:**
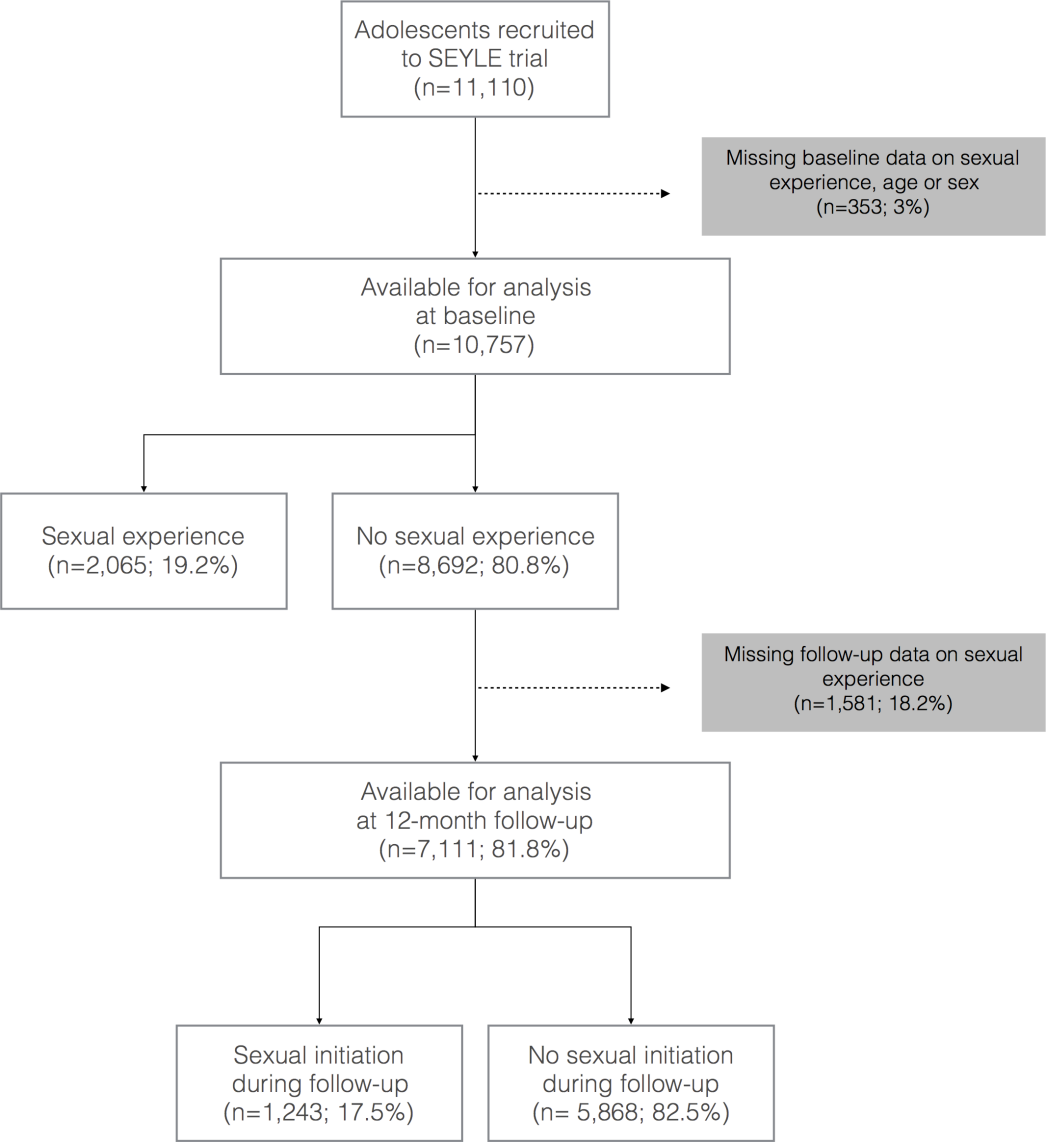
Study flowchart.

Ninety-seven percent of SEYLE trial's pupils (N 10,757; median age 15; interquartile range [IQR] 14–15; females 59.6%) answered a baseline question about sexual experience and were included in a cross-sectional analysis. Follow-up data of the pupils without sexual experience at baseline were analysed through a longitudinal nested case-control design (Fig 1).

### Measurements

The prevalence of baseline sexual experience and sexual initiation during 12-month follow-up were the main study outcomes. Baseline sexual experience was assessed with one yes-or-no question (“Have you ever had sexual intercourse?”). Sexual initiation during follow-up was assessed with a yes-or-no question in the 12-month questionnaire (“During the past twelve months, have you had sexual intercourse?”). Subjects giving an affirmative answer were asked about partners’ number (“During the past twelve months, with how many people have you had sexual intercourse?”) and condom use (“When you have had sexual intercourse during the past twelve months, how often did you or your partner use a condom?”). Those items were recoded as dichotomous variables (1 versus multiple, partners; rarely/never versus always/almost all the time, used a condom). Depending on the report of any of those two factors, a secondary outcome variable with three possible categories was computed: no sexual initiation; sexual initiation, no risk factors reported; risky sexual initiation, one or both risk factors reported.

Several exposure variables were assessed by SEYLE’s baseline questionnaire. Baseline risk behaviours were investigated through the Global School-based Student Health Survey (GSHS) [24]. The following behaviours were defined by dichotomized variables, in agreement with a previous work [1]: excessive alcohol use (≥ twice/week), illegal drug use (≥ three times/lifetime), heavy smoking (> five cigarettes/day), reduced sleep (≤ six hours/night), sedentary behavior (physical activity < once/week), high media use (≥ five hours/day of school/work unrelated use of Internet, television and video-games). Reduced sleep, sedentary behaviour and high media use are referred to as “invisible” risk behaviours throughout the paper, because they may not be perceived as harmful as overt behaviours (e.g. substance abuse) by adult observers [1].

The individual psychological domain was investigated at baseline through the Strengths and Difficulties Questionnaire (SDQ), an internationally validated screening tool which relates well to childhood mental health [25]. The SDQ includes 4 difficulty scales (emotional, peer, conduct, hyperactivity) and a prosocial behaviour scale. In this study, we adopted the broader sub-scale division of SDQ’s difficulty items into internalizing and externalizing scales, which has been recommended for general population samples [26]. The internalizing scale is obtained by combining the emotional and peer items whereas the externalizing scale combines the conduct and hyperactivity items. In analogy with previous research [27], both scales were dichotomized at the 90th percentile, considering as abnormal the measurements above the cut-off. Abnormal externalizing was defined by scores ≥10 for both boys and girls, while abnormal internalizing was defined by scores ≥7 for boys and ≥10 for girls.

Context vulnerabilities were investigated at baseline through measures of problematic interaction with peers, school and family. Trauma exposure was evaluated with SEYLE-specific items [28]. Several bullying manifestations were investigated with yes-or-no questions (“In the past 12 months have others often: spread rumors about you; teased you; deliberately left you out of activities; taken money, property, or food from you; called you names; made fun of how you look or talk”), and a dichotomous variable was created by categorizing any positive answer as being bullied. Physical abuse was investigated with a yes-or-no question about attacks in the previous 12 months. Involvement in fighting was assessed with one question on the number of physical fights in the previous 12 months, and a dichotomized variable was obtained (0 versus ≥1). Truancy was defined as having missed class or school without permission during the previous two weeks. Parental involvement was assessed by asking how often parents/guardians helped making important decisions; took time to talk about things that happened; came to see in case of special activity; paid attention to opinion. These questions had three possible answers (never or almost never; sometimes; often), and were recoded into a new variable where a low parental involvement was defined by two or more “never or almost never” answers.

Age and sex were self-reported at baseline. Age was dichotomized as <16 versus ≥16 years old, because literature shows that, in Europe, sexual debut is infrequently reported by 15 years old [29], and an age of 16 and below is perceived as too young to have sex [30].

### Statistical analysis

Baseline prevalences of risk behaviours, psychological difficulties, trauma, low parental involvement and sexual experience were calculated. The association of age, sex and exposure variables with baseline sexual experience or initiation during follow-up was evaluated through chi-squared tests and simple logistic regression. Age and sex stratified multivariable logistic regression measured the strength of association between potential baseline predictors and the dependent variable sexual initiation (reference category “no sexual initiation”) during the 12-month follow-up. Multiple multinomial logistic regression analyses measured the independent association between predictors and the outcome sexual initiation (reference category “no sexual initiation”), which was subcategorized depending on the absence (Sexual initiation) or presence (Risky sexual initiation) of reproductive risk factors (multiple partners or infrequent condom use). All predictor variables showing a significative association with the outcome at unadjusted analyses were initially included, and only predictors showing significant association in at least one stratum were kept in the final multivariable analyses.

Odds ratios (OR) were calculated together with 95% confidence intervals (CI). Statistical significance of differences was defined by p < 0.05, two-tailed. The analyses were performed with IBM® SPSS® Statistics ver. 23 for Mac OsX.

### Ethics statement

SEYLE was approved by the European Commission, as a precondition for funding, and by the ethics committees of each national centre [21]:

Austria: Ethikkomission der Medizinischen Universität Innsbruck
Estonia: Tallinna Meditsiiniuuringute Eetikakomitee
France: Comité de Protection des Personnes Sud-Méditerranée II
Germany: Ethikkommission Medizinische Fakultät Heidelberg
Hungary: Egészségügyi Tudományos Tanács Titkárság, Pályázati Iroda, Tudományos És Kutatásetikai Bizottság
Ireland: Clinical Research Ethics Committee of the Cork Teaching Hospitals
Italy: Comitato Bioetico di Ateneo, Università Degli Studi Del Molise
Romania: Comisia De Eticã, A Universitãtii De Medicinã Si Farmacie, Cluj Napoca
Slovenia: Komisija Republike Slovenije Za Medicinsko Etiko
Spain: Comité Ètico de Investigación Clinica, regional del Principado de Asturias

An external advisor from the University of Basel, Switzerland, provided independent ethical assessment and supervision of the project [23]. Participation to the project was voluntary and recruitment occurred after obtaining written informed consent from parents/caregivers as well as assent from pupils, in agreement with the guidelines of the involved local ethic committees [23].

### Clinical trial registration

SEYLE was registered in the German Clinical Trials Register (DRKS00000214).

## Results

Experience of sexual intercourse was reported by 19.2% of 10,757 adolescents (Fig 1; Table 1), and was significantly more frequent among pupils ≥16 years old (41% versus 14.2%) and males (20.8% versus 18.1%). The baseline prevalence of sexual experience was also significantly higher among subjects reporting non-sexual risk behaviours, psychological symptoms, trauma, truancy, and low parental involvement (Table 2).

**Table 1:** Baseline characteristics of the study population.

	Respondents N (%)	Overall N (%)	Females N (%)	Males N (%)
*Sexual experience*	10757 (100)	2065 (19.2)	1160 (18.1)	905 (20.8)
*Demographics*				
Age	10757 (100)			
*<16 years old*		8747 (81.3)	5151 (80.3)	3596 (82.7)
*≥16 years old*		2010 (18.7)	1260 (19.7)	750 (17.3)
*Risk behaviours*				
Heavy smoking	10254 (95.3)	1168 (11.4)	695 (11.4)	473 (11.4)
Excessive alcohol	10714 (99.6)	866 (8.1)	389 (6.1)	477 (11.0)
Illegal drugs	10698 (99.5)	511 (4.8)	232 (3.6)	279 (6.4)
Reduced sleep	10355 (96.3)	1622 (15.7)	1109 (17.9)	513 (12.3)
Sedentary behaviour	9724 (90.4)	1137 (11.7)	836 (14.5)	301 (7.6)
High media use	10691 (99.4)	992 (9.3)	518 (8.1)	474 (11.0)
*Psychological difficulties*				
Externalizing	10734 (99.8)	1372 (12.8)	788 (12.3)	584 (13.5)
Internalizing	10738 (99.8)	1277 (11.9)	657 (10.3)	620 (14.3)
*Context vulnerabilities*				
Fighting involvement	10699 (99.5)	2071 (19.4)	649 (10.2)	1422 (33.0)
Physically attacked	10743 (99.9)	1023 (9.5)	379 (5.9)	644 (14.8)
Being bullied	10614 (98.7)	5035 (47.4)	3108 (49.0)	1927 (45.2)
Truancy	10735 (99.8)	1464 (13.6)	777 (12.1)	687 (15.9)
Low parental involvement	10567 (98.2)	1174 (11.1)	695 (11.0)	479 (11.2)

**Table 2:** Correlates of sexual experience at baseline and of sexual initiation during 12-month follow-up.

		Sexual experience at baseline		Sexual initiation during follow-up	
		% ^a^	Odds ratio (95% CI) ^b^	% ^a^	Odds ratio (95% CI) ^b^
*Demographics*					
Age	*<16*	14.2		16.3	
	*≥16*	41.0	4.20 (3.78–4.68)	25.1	1.72 (1.46–2.02)
Sex	*Female*	18.1		17.8	
	*Male*	20.8	1.19 (1.08–1.31)	17.0	0.95 (0.84–1.08)°
*Overt risk behaviours*					
Heavy smoking	*no*	14.2		15.7	
	*yes*	53.7	7.00 (6.15–7.96)	41.2	3.77 (3.05–4.66)
Excessive alcohol	*no*	16.2		16.6	
	*yes*	52.7	5.73 (4.96–6.62)	38.1	3.10 (2.45–3.93)
Illegal drugs	*no*	16.9		17.2	
	*yes*	63.4	8.55 (7.09–10.31)	37.5	2.90 (2.02–4.17)
*Invisible risk behaviours*					
Reduced sleep	*no*	17.0		16.3	
	*yes*	30.6	2.15 (1.91–2.43)	25.5	1.75 (1.48–2.07)
Sedentary behaviour	*no*	18.4		17.5	
	*yes*	22.8	1.31 (1.13–1.52)	18.1	1.04 (0.85–1.28)°
High media use	*no*	18.4		17.5	
	*yes*	26.8	1.62 (1.40–1.89)	17.3	0.99 (0.79–1.23)°
*Psychological difficulties*					
Externalizing	*no*	16.9		16.4	
	*yes*	34.5	2.58 (2.28–2.92)	28.1	2.00 (1.67–2.39)
Internalizing	*no*	18.3		17.5	
	*yes*	25.8	1.55 (1.35–1.78)	17.4	0.99 (0.82–1.21)°
*Context vulnerabilities*					
Fighting involvement	*no*	15.4		16.0	
	*yes*	34.4	2.87 (2.57–3.19)	25.7	1.82 (1.56–2.12)
Physically attacked	*no*	16.8		16.9	
	*yes*	41.5	3.52 (3.07–4.03)	25.5	1.68 (1.35–2.09)
Being bullied	*no*	16.3		15.1	
	*yes*	22.2	1.46 (1.33–1.61)	20.6	1.46 (1.29–1.65)
Truancy	*no*	16.6		16.5	
	*yes*	35.8	2.81 (2.49–3.17)	26.1	1.79 (1.49–2.14)
Low parental involvement	*no*	17.9		16.5	
	*yes*	29.6	1.93 (1.68–2.21)	25.6	1.74 (1.44–2.10)

All p-values are <0.001 except for °>0.05

^a^ Prevalence of sexual initiation depending on demographics and exposure to all considered variables.

^b^ Crude odds ratios and 95% confidence intervals of sexual initiation.

Out of 8692 adolescents without sexual experience at baseline, 7,111 (81.8%; median age 15; IQR 14–15; females 60.7%) completed the 12 months follow-up (Fig 1). Sexual initiation was reported by 17.5% of respondents (1243/7111; mean age 14.84±0.791), without significant difference between girls (17.8%) and boys (17%). Pupils ≥ 16 years old at baseline reported sexual initiation significantly more often than the younger ones (Table 2). Among those having sexual debut during follow-up, 26.4% reported multiple partners (females/males 18.6/35.1%), whereas 17% reported infrequent condom use (females/males 16.6/16.2%). At least one sexual risk behavior was reported by the 37.9% (females/males 33.1/45.6%).

Overt risk behaviours at baseline, such as heavy smoking, excessive alcohol use, illegal drugs use, were the strongest predictors of sexual initiation during follow-up (Table 2), even when adjusting for age and sex (smoking aOR 3.63, 95%CI 2.93–4.50, p<0.001; alcohol aOR 2.95, 95%CI 2.32–3.74, p<0.001; drugs 2.72, 95%CI 1.88–3.92, p<0.001). Sexual debut was also predicted by baseline reduced sleep (age/sex aOR 1.71, 95%CI 1.45–2.03, p<0.001), but not sedentary behaviour or high media use. Externalizing, exposure to fighting and bullying, truancy and low parental involvement were also significantly associated with sexual debut (Table 2).

The association between non-sexual risk behaviours and sexual initiation was more often significant among younger subjects (Table 3). Among older girls, smoking independently predicted sexual initiation. Other independent predictors in subjects ≥ 16 years old were externalizing for girls, and fighting and truancy for boys (Table 3).

**Table 3:** Predictors of sexual initiation during 12 month follow-up in European adolescents: Age and sex stratified analysis.

Independent variables	Sex group	Age group	
		*<16 years old*	*≥16 years old*
Heavy smoking	*female*	2.96 (2.12–4.12) ***	2.30 (1.12–4.73) *
	*male*	2.24 (1.39–3.62) **	2.05 (0.89–4.75) °
Excessive alcohol	*female*	2.54 (1.63–3.96) ***	1.64 (0.76–3.54) °
	*male*	1.91 (1.21–3.02) **	1.43 (0.62–3.28) °
Illegal drugs	*female*	2.87 (1.33–6.18) **	1.12 (0.34–3.74) °
	*male*	1.59 (0.79–3.18) °	1.81 (0.54–6.07) °
Reduced sleep	*female*	1.62 (1.27–2.08) ***	1.01 (0.58–1.77) °
	*male*	1.30 (0.87–1.94) °	0.71 (0.29–1.70) °
Externalizing	*female*	1.37 (1.02–1.84) *	2.60 (1.23–5.49) *
	*male*	1.55 (1.07–2.25) *	0.97 (0.33–2.79) °
Internalizing	*female*	0.85 (0.61–1.18) °	0.76 (0.37–1.55) °
	*male*	0.61 (0.41–0.91) *	0.38 (0.15–0.96) *
Fighting involvement	*female*	1.50 (1.10–2.05) *	1.46 (0.58–3.68) °
	*male*	1.69 (1.31–2.18) ***	2.51 (1.33–4.74) **
Being bullied	*female*	1.42 (1.17–1.73) **	1.43 (0.93–2.21) °
	*male*	1.19 (0.93–1.53) °	1.11 (0.63–1.96) °
Truancy	*female*	1.27 (0.94–1.71) °	0.95 (0.45–1.99) °
	*male*	1.11 (0.76–1.62) °	2.43 (1.07–5.51) *
Low parental involvement	*female*	1.42 (1.05–1.91) *	0.73 (0.33–1.62) °
	*male*	1.55 (1.07–2.25) *	1.07 (0.41–2.78) °

° ≥0.05

* <0.05

**<0.01

***<0.001

Multivariable logistic regression derived odds ratios (and 95% confidence intervals). The dependent variable is “sexual initiation” (reference category “no sexual initiation”). Only predictors showing significant association in at least one stratum were kept in the multivariable analysis.

Girls exposed to substance abuse, reduced sleep, and truancy appeared to have larger odds ratios of risky sexual initiation (versus no initiation) than of sexual initiation without sexual risk behaviours (versus no initiation), although confidence intervals were often overlapping (Table 4). For boys, low parental involvement was significantly associated with risky sexual initiation (versus no initiation) but not to sexual initiation without sexual risk factors. Externalizing independently predicted risky sexual initiation among girls and boys, while internalizing was a protective factor among boys.

**Table 4:** Predictors of sexual initiation with and without associated risk factors (multiple partners, infrequent condom use) during 12 month follow-up.

Independent variables	Sex group	Outcome	
		*Sexual initiation*	*Risky sexual initiation*
Age (<16 years old)	*female*	0.65 (0.50–0.85) **	0.64 (0.44–0.93) *
	*male*	0.62 (0.42–0.92) *	0.43 (0.29–0.65) ***
Heavy smoking	*female*	2.05 (1.42–2.97) ***	4.16 (2.79–6.19) ***
	*male*	2.44 (1.49–4.00) ***	2.09 (1.20–3.62) **
Excessive alcohol use	*female*	2.15 (1.38–3.35) **	2.35 (1.37–4.04) **
	*male*	1.77 (1.08–2.90) *	1.80 (1.06–3.04) *
Illegal drugs use	*female*	2.04 (0.96–4.30) °	2.40 (1.05–5.49) *
	*male*	2.12 (1.08–4.14) *	0.95 (0.38–2.39) °
Reduced sleep	*female*	1.46 (1.12–1.89) **	1.49 (1.05–2.12) *
	*male*	1.18 (0.74–1.87) °	1.08 (0.66–1.78) °
Externalizing	*female*	1.35 (0.98–1.87) °	1.81 (1.22–2.70) **
	*male*	1.35 (0.86–2.12) °	1.59 (1.00–2.52) *
Internalizing	*female*	0.75 (0.52–1.07) °	1.04 (0.66–1.62) °
	*male*	0.55 (0.34–0.89) *	0.57 (0.34–0.95) *
Fighting involvement	*female*	1.46 (1.04–2.06) *	1.57 (1.01–2.44) *
	*male*	1.49 (1.09–2.02) *	2.20 (1.59–3.04) ***
Being bullied	*female*	1.44 (1.17–1.77) **	1.39 (1.03–1.88) *
	*male*	1.30 (0.98–1.74) °	1.07 (0.78–1.46) °
Truancy	*female*	1.01 (0.72–1.41) °	1.73 (1.17–2.54) **
	*male*	1.13 (0.73–1.75) °	1.45 (0.93–2.24) °
Low parental involvement	*female*	1.31 (0.95–1.81) °	1.26 (0.82–1.94) °
	*male*	1.34 (0.85–2.09) °	1.67 (1.05–2.64) *

° ≥0.05

* <0.05

**<0.01

***<0.001

Multivariable multinomial logistic regression derived odds ratios (and 95% confidence intervals). The dependent variable (reference category “no sexual initiation”) was categorized depending on the absence (Sexual initiation) or presence (Risky sexual initiation) of reproductive risk factors (multiple partners or infrequent condom use). Only predictors showing significant association in at least one stratum were kept in the multivariable analysis.

## Discussion

### Key findings

We have identified correlates of adolescents sexual initiation among non-sexual risk behaviours, psychological attributes and social factors, in a large multinational cohort of European adolescents. Baseline sexual experience was reported significantly more often by adolescents ≥ 16 years old and by those exposed to smoking, alcohol and illegal drugs consumption, poor sleep, sedentariness and high media use. Baseline consumption of alcohol, illegal drugs, and tobacco, were strong predictors of sexual initiation during a 12-month follow-up period. Sexual debut was also predicted by baseline reduced sleep, but not sedentary behaviour or high media use (Table 2). The association between risk behaviours and sexual initiation was particularly strong in case of younger females and, among girls, when the outcome was associated with multiple partners and/or infrequent condom use. Significant associations were also observed between sexual initiation and childhood trauma (being bullied, involved in fighting or physically attacked), truancy, and low parental involvement. Externalizing symptoms independently predicted sexual debut, particularly for older girls. Internalizing symptoms were negatively associated with early and risky sexual debut among boys.

### Strengths and limitations

This study included a large sample of European adolescents recruited from randomly selected schools in a large geographic area and ten different countries. Each national sample was population-based and fairly nationally representative. Selection criteria, measurements and procedures were strict and homogenous. The comprehensive sample characteristics, systematically evaluated in SEYLE, allowed to study several exposure variables in the same cohort, including more or less overt risk behaviours, psychological difficulties and problematic interactions with peers, school or family. Another major strength is the longitudinal design, aiming at identifying risk factors which are not simply associated with sexual initiation but also precede it. Additionally, through stratification we could measure disparities between groups of adolescents in relation to their age and sex.

Limitations of the study should be acknowledged. SEYLE only surveyed pupils, and self-report of sensitive data may be biased by social or legal pressure. Nevertheless, participants were thoroughly informed about the strict confidentiality of all procedures, and response rates were high. Another limitation relates to the absence of a definition of the main outcome variable, as respondents may have a different understanding of what “sexual intercourse” is, and adolescents are known to engage in alternative sexual behaviours, such as oral sex. A related limitation is the absence of data on sexual orientation. A normative interpretation of sexual intercourse as “vaginal-penile sex” can overlook sexual minorities, among whom health risks may be higher [31,32]. The questionnaire also lacked a marker for frequency of sex after initiation, which arguably contributes to the health outcomes of adolescent sexuality, as repetitive small life events do [8].

Finally, SEYLE did not collect individual data on socioeconomics, pubertal development, motivations and intentions, all of them being potential correlates of adolescent sexuality [12,33,34].

### Interpretation and implications

Gaining knowledge about adolescents’ risk-taking is challenging because it is common or almost normative, and multiple behaviours often cluster in the same subjects [1,9]. The case of sexual initiation is particularly complex because, apart from being a normative life event, it is also a physiological component of adolescent development. Nevertheless, early sexual activity is associated with reproductive health risk, whose most overt manifestations are unwanted pregnancies and sexually transmitted disease [2–5]. Dangerous health outcomes are commonly mediated by practices which can obviously be correlates of sexuality at any age, although they are particularly prevalent among adolescents [2,5]. In our study, more than one third of adolescents having their sexual debut during 12-month follow-up also reported multiple partners or infrequent condom use.

The association of early sexual initiation with reproductive risk may be interpreted through a gateway model identifying sexuality as an occasion for unsafe sexual behaviour, thus leading to negative health outcomes. As a result, adolescent sexuality is often addressed by downstream individual risk policy responses [3,9] attempting to avoid or delay sexual initiation, or to minimize its potential consequences, through interventions in the reproductive and sexual health domain.

A relevant obstacle to the management of adolescents risk behaviours is that those have been traditionally approached as if belonging to isolated domains of adolescents health [9]. Therefore, our interpretation of interactions between multiple behaviours, as well as its potential to inform policy interventions, is limited. However, data from established surveillance programmes show cross-nationally consistent associations between early sexual activity and overt risk behaviours [10,11]. This is confirmed by our study, as adolescents exposed to alcohol, illegal drugs and smoking reported the highest sexual initiation rates (respectively 52.7%, 63.4%, 53.7%). Gateway interpretations, according to which substance abusers have increased opportunities for sexual risk behaviours [9], are supported by our findings that overt risk behaviours predict earlier and riskier sexual initiation among adolescents, independently of other individual and contextual vulnerabilities.

The gateway-effect interpretation of the association between “invisible” risk behaviors [1], which adult observers may not consider harmful, and sexuality appears more challenging. The association with media consumption may depend from increased exposure to sexual content or opportunity to meet sexual partners [14]. Studies on physical activity have given ambiguous results, as both sedentary behaviour and participation in sports might be associated with early sexual activity [14,35]. However, the only “invisible” risk behavior predicting sexual initiation in our analysis was reduced sleep, a known correlate of adolescents’ development, risk-taking, sedentariness, and high media use [1,36–38].

Although the interest in evaluating the coexistence of risk behaviours is justified by gateway hypotheses and the possible incremental physical health outcomes, adolescent sexual behaviours also have potential effects on mental and social health domains of wellbeing, which are arguably mediated by the evaluation that young people make of their own behaviours [8]. A distinctive feature of the present study is the evaluation of several individual and contextual factors which may influence adolescents’ engagement in risk behaviours as well as their perception of the same behaviours. For instance, age and sex related disparities should not be overlooked. The significantly low rate of sexual initiation among subjects younger than 16 years old (14.2% versus 41% in those ≥16) was expected [10,39]. European adolescents’ median age at sexual debut is 17 years, while less than 15% become sexually active before the age of 15 [29]. In addition, despite legal ages of consent variating between 14 and 16, European teenagers and parents consider people under 17 years old to be “too young to have sexual intercourse”, and higher age norms among parents are negatively associated with early debut [30]. It is not even surprising that less girls than boys reported sexual activity at baseline, as previously described at European level [12,29], although the gap is inconsistent across countries and becomes narrower with age, as shown by our longitudinal data. Interestingly, overt risk behaviours such as substance abuse, which are in general more common among boys [1], were strongly predictive among younger girls. Additionally, a strong association between non-sexual risk behaviours and risky sexual initiation was suggested among females. These findings are consistent with previous studies [30,40] and may partially be explained by sexual norms, stigma and double standards, whose consequences on sexual behaviours and their perception-mediated outcomes are often worse among women [20]. It therefore seems plausible that differences between girls and boys are not limited to their timing or level of engagement in specific behaviours but extend to the trajectories between risk factors and outcomes, which are arguably modulated by individual and contextual vulnerabilities [41,42].

For instance, adolescent psychological difficulties are related to risk-taking patterns [1,17]. Abnormal internalizing and externalizing symptoms are more prevalent among adolescents engaging in multiple risk behaviours [1], and both were significantly associated with sexual initiation in our study. Externalizing is common in undercontrolling adolescents who engage in overt risk [1,41] and we found that it predicts sexual debut in girls and boys, independently of other risk behaviours. On the contrary, internalizing, which is particularly frequent among adolescents with sedentary behaviour and high media consumption [1], was not predictive for girls whereas it was negatively associated with sexual initiation during follow-up among boys. The positive baseline association may therefore be age-specific or even depend on an inverse directionality between sexual activity and internalizing symptoms.

Our study also evaluated the interaction between adolescents and their social context, including peers, school, and family, which previous research has highlighted as source of both risk and protective factors [4,16,43–45]. Bullying victimization is associated with self-injurious behaviour though peer and parental support have a protective role [45]. Childhood trauma and violent behaviors, whether as victims or perpetrators, are related to adolescent mental health and sexual activity [15,29,39]. It should also be reminded that forced sexual initiation, despite being relatively infrequent in developed countries, is significantly more prevalent among individuals reporting an earlier debut and females [46,47]. Our study could not specifically address exposure to non-consensual sex or sex-related trauma though it shows a significant association between fighting, being bullied, or low parental involvement, and earlier sexual initiation. The protective potential of an inclusive, vigilant and caring social context is further supported by the findings that risky sexual behaviour was predicted by low parental involvement among boys and by truancy among girls.

In conclusion, the practice and research implications of our findings share reservations about single-domain perspectives on adolescent risk behaviour and health. Responses which are limited to the reproductive health domain overlook cross-domain interactions that could guide the early identification of risk subjects and the development of preventive initiatives. Further studies of the relationship between adolescent sexual and non-sexual risk behaviour, also in look of possible reverse gateway effects, may result in useful information to downstream policies. However, it seems simplistic to reduce any association between risk behaviours and early sexual initiation to a gateway effect, which overlooks the role of individual and contextual factors in determining behaviours and modulating their effect on physical as well as mental and social health [8,9,17,18]. Refuting simplified gateway hypotheses in favour of a more comprehensive model would therefore be particularly valuable, in order to achieve a deeper understanding of sexual behaviours and their potential health outcomes, and to promote the positive effects of sexuality on adolescent wellbeing [8]. Resulting research and policies would respectively study and implement tailored interventions targeting adolescent health as a whole instead of focusing on limited health outcomes. Special consideration may, for instance, be needed for younger girls because the strong association between non-sexual and sexual behaviours identifies them as a particularly vulnerable group, in view of double standards and distinctive reproductive and gynaecological health risks.
